# Toll-Like Receptor 4 (TLR4) and AMPK Relevance in Cardiovascular Disease

**DOI:** 10.34172/apb.2023.004

**Published:** 2021-10-10

**Authors:** Haleh Vaez, Hamid Soraya, Alireza Garjani, Tooba Gholikhani

**Affiliations:** ^1^Department of Pharmacology and Toxicology, Faculty of Pharmacy, Tabriz University of Medical Sciences, Tabriz, Iran.; ^2^Department of Pharmacology, Faculty of Pharmacy, Urmia University of Medical Sciences, Urmia, Iran.; ^3^Student Research Committee, Faculty of Pharmacy, Tabriz University of Medical Sciences, Tabriz, Iran.; ^4^Nanora Pharmaceuticals Ltd, Tabriz, Iran.

**Keywords:** AMPK, Cardiovascular disease, Inflammation, TLRs

## Abstract

Toll-like receptors (TLRs) are essential receptors of the innate immune system, playing a significant role in cardiovascular diseases. TLR4, with the highest expression among TLRs in the heart, has been investigated extensively for its critical role in different myocardial inflammatory conditions. Studies suggest that inhibition of TLR4 signaling pathways reduces inflammatory responses and even prevents additional injuries to the already damaged myocardium. Recent research results have led to a hypothesis that there may be a relation between TLR4 expression and 5' adenosine monophosphate-activated protein kinase (AMPK) signaling in various inflammatory conditions, including cardiovascular diseases. AMPK, as a cellular energy sensor, has been reported to show anti-inflammatory effects in various models of inflammatory diseases. AMPK, in addition to its physiological acts in the heart, plays an essential role in myocardial ischemia and hypoxia by activating various energy production pathways. Herein we will discuss the role of TLR4 and AMPK in cardiovascular diseases and a possible relation between TLRs and AMPK as a novel therapeutic target. In our opinion, AMPK-related TLR modulators will find application in treating different immune-mediated inflammatory disorders, especially inflammatory cardiac diseases, and present an option that will be widely used in clinical practice in the future.

## Introduction

 Inflammation has long been associated with many diseases, including diabetes, cancer, arthritis, and cardiovascular complications. It is triggered when the immune system, including bone marrow-derived cells like monocytes and macrophages and non-bone marrow-derived cells, detects infection or tissue injury as the body’s first-line defense. Since TLRs are expressed in almost all heart cells, their involvement in developing cardiovascular diseases (CVDs) is evident. However, recent regard to the importance of innate immunity leads to new investigation areas. It may result in identifying potential novel therapeutic targets for inflammatory diseases treatment.

 Toll-like receptors (TLRs) are essential receptors of the innate immune system, prerequisites for inducing adaptive immune responses against pathogens. They have been termed pattern recognition receptors that detect conserved motifs on pathogens, pathogen-associated molecular patterns (PAMPs), lipopolysaccharide (LPS) of gram-negative bacteria, or viral double-stranded RNAs.^1^ Furthermore, TLRs can also recognize damage-associated molecular patterns or danger-associated molecular patterns (DAMPs), which are endogenous host material propagated during cellular stress or death.^2^ Thereby, TLRs play a role in different steps of recognizing through innate immunity and regulate the generation of adaptive immunity by forming immunological memory and polarization of antigen responses and responses to tissue damage.^3^

 Although inflammation is a crucial survival mechanism, it can induce severe damage to the tissue in case of being inordinate or turned into a chronic state. Hence, the identification and studying the immune regulators is of value. The 5’ adenosine monophosphate-activated protein kinase (AMPK) is a novel immune modulator with therapeutic efficacy in different inflammatory conditions.^4,5^ There are several publications describing the role of AMPK activation in inhibiting inflammatory responses induced by different stimulating factors like acute and chronic colitis,^6^ cystic fibrosis,^7^ autoimmune encephalomyelitis,^8^ and pro-inflammatory effects after lung injury.^9^ The results of several studies suggest a hypothesis that there may be a relation between AMPK and TLR4 signaling in various inflammatory conditions.^10-14^ This review will summarize the role of TLR4 in CVDs and elaborate on the hypothesis that the AMPK involve in improving the immune responses following tissue injury and the inflammatory process.

## TLRs

 The Toll protein was first discovered as the receptor responsible for early embryonic development in Drosophila fruit fly.^15,16^ Medzhitov et al^17^ explained that the human homolog of Drosophila Toll protein could trigger an innate immune response. To this day, 11 human and 13 mice TLRs have been identified. TLR-1, TLR-2, TLR-4, TLR-5, TLR-6, TLR-10, TLR-11, and TLR-12 are expressed on the cell surface and recognize components derived from microbes such as lipids, lipoproteins, and proteins. Whereas TLR-3, TLR-7, TLR-8, TLR-9, and probably TLR-13 of mice are intracellular proteins that detect nucleic acids. TLRs 11 and 13 are only expressed in mice.^18^

 TLRs are expressed in various types of tissues, including the heart, brain, kidney, liver, lung, small intestine, spinal cord, spleen, and reproductive organs.^19^ Since among all TLRs, TLR4 is the most expressed protein in the heart, as a potential and significant receptor has been extensively investigated for its critical role in different myocardial inflammatory conditions, including endotoxemia,^20,21^ myocarditis,^22^ myocardial infarction (MI),^23^ ischemia/reperfusion (I/R) injuries,^24^ heart failure,^25^ aortic valve diseases,^26^ atherosclerosis,^27^ and hypertension.^28^

## TLR signaling

 TLR4 is well known as the receptor for LPS, a gram-negative bacteria membrane component of the outer membrane.^29^ Fusing of TLR4 with myeloid differentiation 2 (MD2) on the cell surface is essential for ligand-induced signaling activation.^30^ Several proteins modulate TLR signaling pathways that improve LPS recognition: LPS-binding protein (LBP) and CD14, a glycophosphatidylinositol-anchored protein.^31,32^

**Figure 1:**
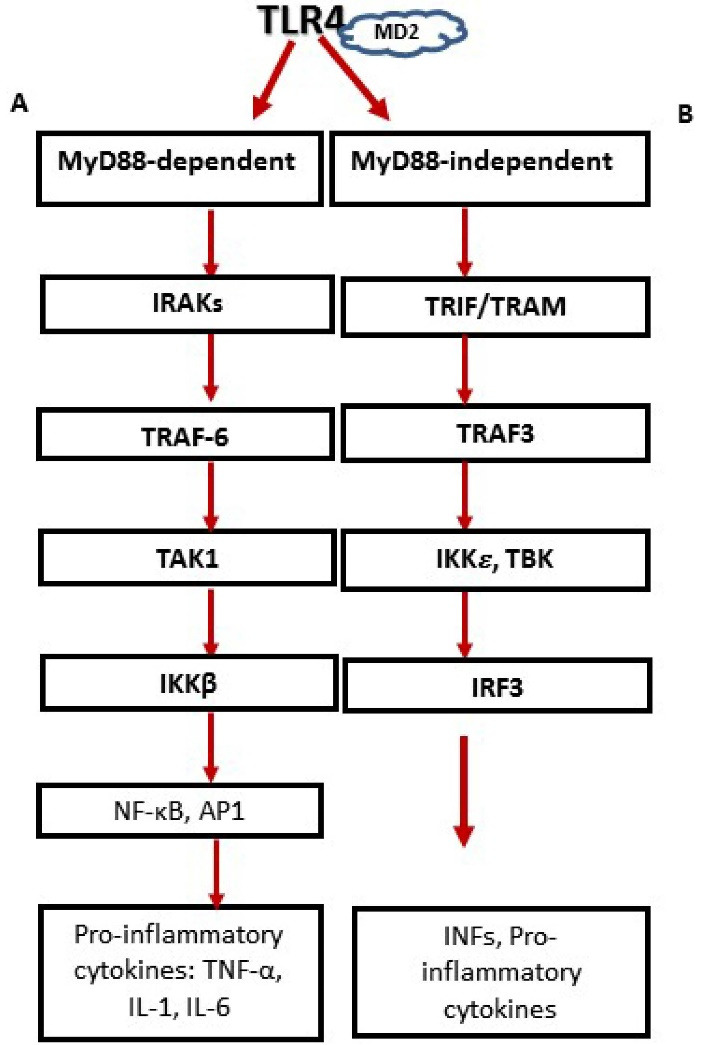
Two distinct signaling pathways after TLR4 activation. MyD88-dependent and the MyD88-independent pathways. (**A) **MyD88 engages IL-1 receptor-associated kinases (IRAKs), followed by interplay with TNF-receptor-associated factor 6 (TRAF6), and the downriver stimulation of transforming growth factor 𝛽-activated kinase 1 (TAK1). Then, activated TAK1 phosphorylates IkB kinase complex (IKKβ), directing the phosphorylation and degradation of I-κB, which discharge nuclear factor-κB (NF-𝜅B) and results in the nuclear translocation and DNA attaching of NF-κB where it stimulates multiple gene expression.** (B)** MyD88-independent pathway involves the recruitment of adaptor proteins TRIF and TRAM, and the evocation of IFN regulatory factor 3 (IRF3) nuclear translocation, facilitated by TBK1 (tank-binding kinase 1) and IKK𝜀 ( inhibitory κB (IκB) kinase ε) which finally result in production of type I IFNs and proinflammatory cytokines.

 Upon activation and ligand recognition, TLR4 induce the assembly of homodimerization. Depending on the adaptor usage, the intracellular signaling pathways are divided into two pathways as shown in Figure 1: the myeloid differentiation factor 88 (MyD88)- dependent and the MyD88-independent pathways.^33^

 MyD88 associates with all TLR types except TLR3. MyD88 engages IL-1 receptor-associated kinases (IRAKs), followed by interaction with TNF-receptor-associated factor 6 (TRAF6), and the downstream stimulation of transforming growth factor 𝛽-activated kinase 1 (TAK1), facilitated by the adaptor proteins, TAK1-binding protein 2 and TAK1- binding protein 3 (TAB2 and TAB3). Then, activated TAK1 phosphorylates IkB kinase complex (IKKβ), directing the phosphorylation and degradation of I-κB, which discharge nuclear factor-𝜅B (NF-𝜅B) and result in the nuclear translocation and DNA attaching of NF-κB where it stimulates multiple gene expressions.^34^

 MyD88-independent pathways or Trif-dependent pathway (Toll/interleukin-1 receptor-like (TIR) domain-containing adaptor inducing IFN-β) is utilized by TLR3 and TLR4. It involves the recruitment of adaptor proteins TRIF and TRAM (TRIF-related adaptor molecule), activation of TNF receptor-associated factor 3 (TRAF3), and the evocation of IFN regulatory factor 3 (IRF3) nuclear translocation, facilitated by tank-binding kinase 1 (TBK) and IKK𝜀 which finally result in the production of type I IFNs.^18,35^

## TLRs and inflammation

 The production of inflammatory cytokines and chemokines directly affects TLRs stimulation upon ligand encountering, which directs various inflammatory cells into the ligand presented sites. The appropriate inflammatory response induced by TLRs is required to function both innate and adaptive immune responses. However, its inordinate signaling may result in autoimmune/inflammatory diseases.^36^ Thereby, TLR signaling needs to be strictly adjusted.

 Cells have obtained a series of strategies for negative regulation of TLR signaling at multiple steps. Complex regulation at several levels varying from extracellular soluble TLRs to transmembrane and intracellular inhibitors such as those that retain the TIR domain indicates that regulation can be acquired by a cascade of regulators. This declares that a specific inhibitor may be integral but not sufficient to adjust the pathway.^37,38^

 A negative regulator of TLR signaling is a transcriptional repressor, the orphan nuclear receptor small heterodimer partner (SHP), which abundant LPS-induced expression of its lead-in inhibition of TLR signaling in AMPK dependent pathway.^39^

## TLR4 and CVDs

 Besides immune cells, TLRs are expressed in endothelial cells, smooth muscle cells, and cardiomyocytes.^40^ As previously mentioned, TLR4 is the most studied TLRs with the highest expression level in the heart. Although cardioprotective effects of short-term activation of this receptor have been demonstrated in isolated perfused hearts,^41^ studies have reported its implication in myocarditis, MI, myocardial I/R injury, heart failure, cardiac arrhythmias, cardiac valve diseases, atherosclerosis, and hypertension.^40,42^ TLR4 is involved in the etiology of hypertension and introduced as a possible novel therapeutic target for hypertension treatment. Recent studies have been suggested a cross-talk between angiotensin II (Ang II) and TLR4 in hypertension.^43^ It was reported that Ang II upregulated the TLR4 on mesangial cells and finally contributed to renal inflammation and fibrosis.^44,45^ Zhang et al^46^ demonstrated that TLR4 antagonist, TAK-242, reversed aldosterone-induced cardiac and renal injury. An increased level of TLR4 expression in advanced heart failure has been reported previously, and inhibition of this receptor reduced the heart failure progression.^40,47^ Cardiomyocyte necrosis due to MI releases various endogenous DAMPs associated with profound TLR4 activation and inflammatory responses, leading to additional damage to the injured myocardium.^48^ Soraya et al^13^ reported cardioprotective effects through suppression of TLR4 expression in MI.

## AMPK

 AMPK, a serine/threonine kinase, is a highly conserved sensor of cellular energy balance and is activated under the condition of ATP depletion induced by any cellular stresses such as hypoxia, glucose deprivation, or metabolic prevention of ATP biogenesis, exercise, osmotic stress, and oxidative stress.^49^ AMPK consists of catalytic α units and β and γ regulatory units that jointly make a heterotrimeric functional enzyme. The multiple AMPK subunit isoforms encoded by distinct genes are expressed in different tissues such as the liver, brain, and skeletal muscle and therefore have a tissue-specific distinct distribution.^50,51^ Among the seven isoforms of AMPK, the α_2_ and β_2_ isoforms are significantly expressed in the heart. Each subunit isoform’s differential distributions provide a tissue-selective regulation of the AMPK activity.^52^

 AMPK activation occurs by an increase in the concentration of AMP and its binding to the γ subunit, which induces a conformational alteration in the AMPK structure, and allosterically actuating assembly to the α catalytic subunit, triggers phosphorylation of the Thr172 residue by upstream AMPK kinases, and prevents the effect of protein phosphatase 2C from dephosphorylating Thr172.^53^

 Some clinically used drugs pharmacologically activate AMPK in conventional medicine, derived from natural sources in traditional medicines, food, or beverages.^54^ 5-Aminoimidazole-4-carboxamide riboside (AICA riboside), an adenosine analog, mimics AMP’s effects on the AMPK complex and activates AMPK in most cells.^55^ Metformin, phenformin, and glargine increase the cellular ADP: ATP ratio and activate AMPK by inhibiting Complex I of the respiratory chain.^56^ The anti-diabetic medicines of thiazolidinediones class activate AMPK both by a humoral, adiponectin-dependent procedure and through a cell-autonomous, adiponectin-independent mechanism.^54^ 2-Deoxyglucose (2DG), as a repressor of glycolysis, activates AMPK partially by restoring ATP, causing an increase in ADP: ATP due to its phosphorylation by hexokinase.^56^ A-769662, a thienopyridine compound, mimics AMP’s effects on the AMPK system, i.e., allosteric activation and protection against Thr-172 dephosphorylation.^57^ The natural ligand of salicylate binds at a position that interferes with that used by A-769662. It is considered a direct activator of AMPK with less potency than A-769662.^58^ Phenobarbital,^59^ plant products like resveratrol,^60^ epigallocatechin-3-gallate,^61^ curcumin,^62^ garlic oil,^63^ berberine,^64^ hispidulin^65^ can also activate AMPK. PT1, a small molecule activator of AMPK, promotes ACC phosphorylation without altering the AMP: ATP ratio.^66^ Inhibition of AMP-metabolizing enzymes like phosphodiesterases, by inhibitors like sildenafil, may also be considered as AMPK activators.^67^

 Accumulating evidence from *in vitro* and *in vivo* experiments indicates the involvement of AMPK as a negative regulator of immune/inflammatory responses, and shaping the cell’s metabolic status at sites of inflammation may serve as a novel and promising therapeutic approach in the pathophysiology of several immune-mediated inflammatory diseases. The anti-inflammatory effects of AMPK activators have been shown to confer benefits in colitis,^6,68^ hepatitis and liver disturbances,^69,70^ autoimmune encephalomyelitis,^71^ osteoarthritis,^72^ endotoxemia,^73-75^ MI,^76^ angiogenesis,^77^ acute kidney injuries,^78^ and mouse lung injury model.^79^

**Figure 2:**
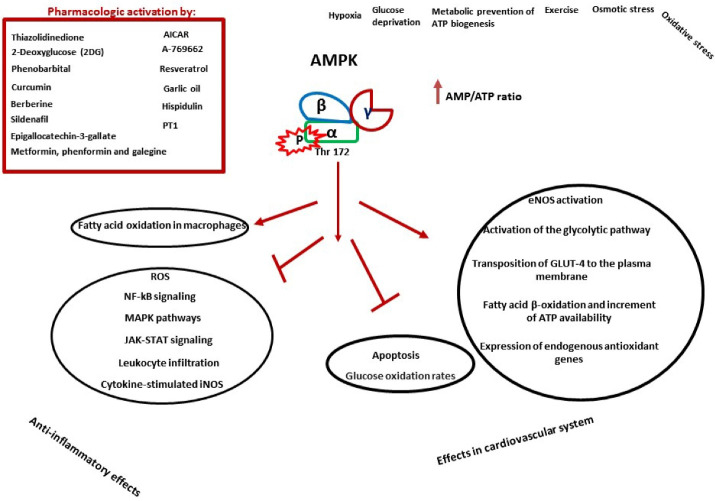
AMPK is phosphorylated and activated by various agents. These factors can be energy depleting conditions (such as hypoxia, metabolic prevention of ATP biogenesis, etc.) or pharmacological agents (for example: metformin, A-769662, etc.). After phosphorylation in threonine 172 α subunit, different pathways are activated. AMPK activation demonstrate anti-inflammatory effects and also ambivalent effects on the cardiovascular system. Some of the main anti-inflammatory mechanisms of AMPK are through inhibition of NF-κB signaling, MAPK pathways, JAK-STAT signaling, leukocyte infiltration, cytokine-stimulated iNOS and increased fatty acid oxidation in macrophages. In the cardiovascular system, activation of AMPK can reduce glucose oxidation and apoptosis, on the other hand, it can increase fatty acid oxidation, GLUT-4 transport to the plasma membrane, glycolysis, eNOS activation and expression of antioxidant genes. AICAR: 5-Aminoimidazole-4-carboxamide riboside; PT1: AMPK activator; ROS: reactive oxygen species; GLUT-4: glucose transporter 4; eNOS: endothelial isoform of NO synthase. **→**: Stimulation/Increase; ┤: Inhibition/Decrease.

 AMPK is consistent with the suppressed production of pro-inflammatory mediators and the promotion of anti-inflammatory cytokines.^80^ Some of the main molecular anti-inflammatory mechanisms of AMPK were reviewed by Salt and Palmer^81^ in 2012 and shown in Figure 2, which include inhibition of cytokine-stimulated iNOS (inducible NO synthase) protein expression and inhibition of NF-kB signaling,^82^ inhibition of MAPK pathways,^83^ modulations of reactive oxygen species (ROS) synthesis,^84,85^ inhibition of JAK-STAT signaling,^86^ prevention of leukocyte infiltration,^87^ regulation of cytokine synthesis^88^ and regulation of lipid metabolism by maintaining fatty acid oxidation in macrophages to limit inflammation.^89^

## AMPK and CVD

 AMPK has various implications of great importance in the cardiovascular system. While AMPK signaling has a particular physiological role in the heart cells like other tissues, its importance relies on its activation by stress conditions like an extra hemodynamic burden, myocardial ischemia, and hypoxia. Under the mentioned conditions, the energy metabolism role of AMPK is to maintain sufficient ATP levels by improving the uptake of glucose by transposition of glucose transporter 4 (GLUT-4) into the cardiomyocytes,^90^ activation of the glycolytic pathway by phosphorylating phosphofructokinase-2, augmentation of fatty acid β-oxidation, and increasing ATP availability.^91-93^

 AMPK induces endothelial isoform of NO synthase (eNOS) activation,^94^ which leads to the generation and release of NO from the endothelium. NO as an endothelial protective agent play a role by the promotion of vascular smooth muscle relaxation and proliferation, inhibition of leukocyte adherence and migration, platelet aggregation, and production of adhesion molecules.^95^

 Different studies reviewed by Shirwany and Zou^93^ in 2010 suggest a complicated relation between the AMPK cascade and the redox ratio in cardiovascular homeostasis. This correlation emerged to be free from its assumed function energy-conserving pathways in the cell. AMPK regulates the antioxidant system by expressing endogenous antioxidant genes and repressing oxidants’ synthesis into cell survival.^93^

 Plenty of studies have been monitoring the relation of AMPK with the different pathophysiological status of the cardiovascular system. They indicated that AMPK can modulate the cardiac hypertrophy procedure,^96^ potentiate preconditioning effect in the ischemic heart,^97^ involves in the pathogenesis of the Wolf-Parkinson-White syndrome in case of mutations in the γ2 subunit of AMPK,^98^ plays as a prominent constitute in angiogenesis and neointimal hyperplasia and also unclarified traces of this enzyme was observed in atherosclerosis development.^99^

 AMPK, through regulating ATP synthesis and consumption controls energy metabolism, is assumed to be a promising target for heart protection ischemic injuries.^100^

 In the ischemic condition of the myocardium, increased rates of fatty acid oxidation consequently inhibit glucose oxidation. Also, accelerated glycolysis during ischemia increases lactate production and intracellular acidosis by promoting calcium overload during the reperfusion period. As an essential regulator of fatty acid oxidation, malonyl-CoA prevents the uptake of fatty acids into the mitochondria. AMPK phosphorylates and inhibits acetyl-CoA carboxylase (ACC), suppressing the synthesis of malonyl-CoA. Furthermore, it phosphorylates and activates malonyl-CoA decarboxylase (MCD), increasing the degradation of malonyl-CoA levels, resulting in a decreased rate of glucose oxidation.^101,102^

 Fatty acid oxidation of AMPK may attenuate glucose oxidation, leading to acidosis due to increased lactate and proton production. This influence cardiac efficiency and result in the severity of ischemic injuries. In this case, the prohibition of the ischemic-induced activation of AMPK and suppressing the downstream decline in the malonyl-CoA ratio support the notion that it may be a novel and beneficial therapeutic strategy to decrease ischemic heart disease outcomes.^102^

 On the other side, there is a critical role of AMPK activation in glucose uptake and ATP production, especially in the energy deprivation status of ischemia, through translocation of the glucose transporter 4 from intracellular compartments the cell surface.^103^ Furthermore, apoptotic procedure induced by ischemia and infarction increased in AMPK-deleted mice,^104,105^ expressing the importance of AMPK as a double-edged sword.

## AMPK and TLR4 relation

 The correlation between AMPK and TLR4 is a novel research topic, with various unknown aspects; different studies have mentioned this and tried to determine how the relationship exists. A study showed that aerobic exercise could improve insulin resistance, blood glucose level, and lipid profile in diabetic mice. The authors attributed the beneficial effects to the TLR4 mediated ERK/AMPK signaling pathway in this study. Reporting that, the upregulation of TLR4 expression level increases ERK and AMPK activity.^106^ Another study showed that dihydroquercetin reduced inflammation in LPS-induced endotoxemia through enhancement of AMPK phosphorylation and downregulation of TLR4 expression.^75^ Another link of AMPK and TLR4 were documented in hypothalamic pharmacological activation of AMPK (hyp-AMPK) in LPS-treated mice. It was reported that hyp-AMPK activation suppressed the effects of LPS on the hyp-AMPK phosphorylation, liver PEPCK expression, and glucose generation in a TLR4-dependent manner because none of these changes were affected in TLR4-mutant mice. These findings demonstrated the relation between hypothalamic AMPK dephosphorylation and TLR4 protein in hypoglycemia evoked by LPS. Although TLR4 expression in the hypothalamus was not modified in LPS-treated mice, on the contrary, transforming growth factor b-activated kinase 1 (TAK1) phosphorylation and TLR4/MYD88 association were increased.^12^ TLR and AMPK relation in reducing adipogenesis, adipose inflammation, and promoting energy expenditure recently was confirmed in a mice model of obesity by modulating SIRT1 (Sirtuin 1, an enzyme function as intracellular regulatory proteins), TNF-α and IL-6 associated metabolic and inflammatory pathways.^107^

 Despite numerous references to the anti-inflammatory role of AMPK, the pro-inflammatory impress of AMPK in TLR4 cascade was mentioned by Kim et al^108^ in 2012. They demonstrated that AMPK-α_1_ has an essential upstream kinases role in stimulating pro-inflammatory signals via the activation of TAK1. The deficiency of AMPK-α_1_ or inhibition of AMPK-α_1_ activity by compound C consequence in the dramatic decrease in TAK1 activity, subsequent blocking of downstream signaling cascades, and the expression of NF-kB-dependent genes in response to LPS stimulation, pointing out to an AMPK-α_1_-TAK1-NF-kB axis in TLR4-mediated signaling. One of the valuable studies that can help clarify the relationship between TLR4 and AMPK was the research done by Kim et al.^109^ On the evaluation of anti-inflammatory effects of Andrographolide, a well-known labdane diterpenoid of Andrographis paniculata. Andrographolide significantly reduced LPS-induced pro-inflammatory cytokines by suppressing NF-κB, MAPK, and their upstream signaling pathways through activating the AMPK. They showed that activation of AMPK could inhibit ERK/JNK/p38 pathway. Simultaneously, directly interact with TAK1, inhibit TAK1-dependent MAPK signaling cascade, and subsequently inhibit NF-kB induced pro-inflammatory cytokine genes.

 The other link that turned light in determining TLR4 and AMPK relation reported by Yuk et al.^39^ They showed that activation of NF-κB via LPS-induced TLR signaling evoked SHP transcription via a CaMKKβ–AMPK–USP1-dependent signaling pathway that eventually suppressed NF-κB in a negative feedback loop. It is demonstrated that AMPK, via the Nrf2/antioxidant responsive element (Nrf2/ARE) signaling pathway, stimulated the heme oxygenase (HO)-1 system, which diminished cellular stress and inhibited TLR signaling.^110^ Severe inflammation have been demonstrated in HO-1 knockout mice and in a human case of genetic HO-1 deficiency, which showed the significant anti-inflammatory role of the HO-1/CO system.^111^ Upon AMPK activation, it stimulates SIRT1, Peroxisome proliferator-activated receptor-γ coactivator (PGC)-1α, Forkhead transcription factor (FoxO), and p53, which could attenuate NF-κB activation. AMPK is a master controller of PGC1α, a transcriptional coactivator described as a master regulator of mitochondrial gene expression, including oxidative phosphorylation and ROS detoxification, and is associated with the pathogenesis of the CVD. PGC-1α and NF-κB are mutually regulated during inflammation, where oxidative stress plays an essential role.^112^ FoxOs are phosphorylated and activated by AMPK. Many investigators have demonstrated that FoxO plays a crucial role in inducing various downstream genes involved in oxidative stress response and suppressing ROS. FoxO6 suppresses pro-inflammatory gene up-regulation by NF-κB, and thus reducing oxidative stress.^113^

**Figure 3:**
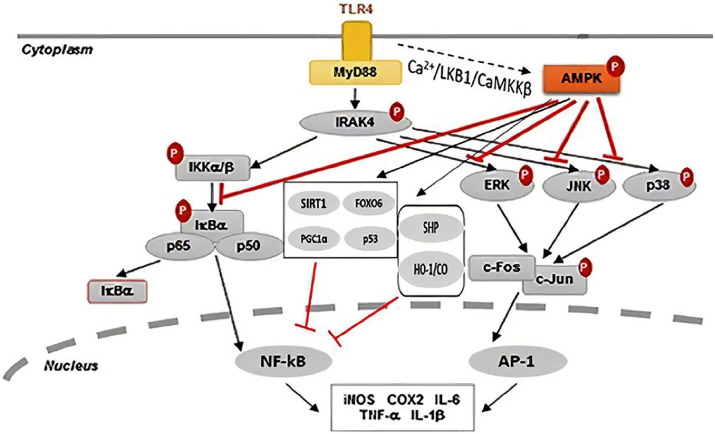
AMPK can ameliorate inflammatory responses via various direct and indirect anti-inflammatory signaling pathways. Engagement of TLRs by DAMPs activates inflammatory responses and upon TLR4 stimulation, the activation of AMPK can be simultaneously induced by LKB1 and Ca^2+^-calmodulin-dependent protein kinase. Activation of AMPK can inhibit ERK/JNK/p38 pathway. Simultaneously, directly interact with TAK1 and inhibit TAK1-dependent MAPK signaling cascade and subsequently inhibit NF-κB-induced pro-inflammatory cytokine genes. ↓, activation; ⊥, inhibition. AMPK, AMP-activated kinase; AP-1, activator protein 1; CaMKKβ, Ca^2+^/calmodulin-dependent protein kinase kinase-β; FoxO, Forkhead transcription factor; HO-1/CO, heme oxygenase-1/carbon monoxide; PGC-1α, Peroxisome proliferator-activated receptor-γ coactivator; SHP, small heterodimer partner; SIRT1, Sirtuin 1; TLR, Toll-like receptor.

 The various mechanisms in the TLR4-inflammatory pathway which can undergo modulation by AMPK are demonstrated in Figure 3. However, it should be noted that the role of AMPK in inflammatory pathways is complicated and may be conflicting depended on specific cell types.

## AMPK and TLR 4 relation in the cardiovascular system

 In 2012, it was confirmed that acute treatment with metformin improves ECG pattern and cardiac function following isoproterenol-induced MI.^114^ After this study, it was indicated that short-term administration of metformin in MI, particularly in an AMPK activating dose, profoundly suppressed post-MI remodeling and pro-inflammatory reactions, as demonstrated by a decline in the myeloperoxidase activity in the myocardium and a reduction in the TNF-α and IL-6 content in the heart tissue and serum. These results were correlated with the suppression of the mRNA level of TLR4 in the cardiac tissue and the diminution of the protein level of MyD88, which were elevated in the myocardium following isoproterenol-induced MI.^13^ This finding of AMPK activation by metformin and the subsequent inhibition of TLR4 expression and activity led to the hypothesis that there may be a link between AMPK and TLRs. The importance of AMPK as a cardioprotective agent against ischemic injury of the heart also was suggested in a model of deficiency in TLR4 signaling. It was shown that in regional ischemia induced in wild type c3H/HeN and *Tlr-4* mutated C3H/HeJ mice, elimination of *Tlr-4* had a cardio-protective outcome on ischemic damages due to activation of AMPK and ERK signaling pathways. It was explanatory that TLR4 is negatively correlated to AMPK activation in the heart following ischemia-reperfusion.^14^ Also, Fang et al^115^ indicated that the immune reaction processes are interceded by the TLR4 signaling cascade (MyD88-dependent pathway and TRIF-dependent pathway) interfacing with other pathways (PI3K/Akt and AMPK, ERK signaling pathway). Investigation on modulating fatty acid metabolism after diabetes declared that although diabetes increased AMPK phosphorylation in both non-obese diabetic mice and TLR4-deficient non-obese diabetic mice, however, TLR4-deficient group demonstrated a higher level of AMPK and acetyl-CoA carboxylase phosphorylation. Thereby, it was speculated that TLR4 deletion might boost the upregulation of cardiac fatty acids metabolism via AMPK activation following diabetes and indicate a potentially significant relation of TLR4 and AMPK in the progression of diabetic cardiomyopathy and dysfunction.^116^

 A study showed that high-mobility groupbox 1 (HMGB1) plays a pathological role in doxorubicin-induced heart failure via TLR4. They showed that inhibition of HMGB1 exerts cardioprotective effects by increasing AMPK protein level and preventing cardiomyocyte apoptosis.^117^ Concerning this, it was reported that TLR4 engagement inhibits AMPK activation through an HMGB1 protein-dependent mechanism in LPS-induced acute lung injury.^118^

 Recently, in one of our published studies, we showed that metformin, as an AMPK activator, protects the lung tissue from LPS-induced acute injury through activation of AMPK and suppression of TLR4.^20^ This pointed to the fact that the linkage of TLR4 and AMPK is not limited to cardiac tissue. Therefore the beneficial effects of metformin in cardiac activity in septic crisis may be due to other body reflexes, including anti-inflammatory roles in other organs such as the lung. To examine this hypothesis, we performed the isolated heart modeling of sepsis by using LPS. What was acquired rejected this supposition, because again, metformin suppressed TLR4 expression via the AMPK-dependent pathway. It pointed to the significance of TLR4-involved local immune reactions in the LPS-induced myocardial dysfunction. It inferred a clear connection between AMPK and TLR4 even far from the systemic circuit and circulatory immunity.^22^ Using metformin in sepsis induced myocardial dysfunction, reduced TLR4 gene expression and decreased the protein content of MYD88 and TNF-α level in the heart. Interestingly, these effects again were AMPK-dependent, and inhibition of AMPK by compound C reversed the influence of metformin in this endotoxemic model.^119^

 A study by Sun et al^120^ demonstrated that vaccariae hypaphorine (VH), the main active compound of the *Vaccaria segetalis, *reduced LPS-induced inflammatory cytokine production in human endothelial EA·hy926 cells. In this study, pre-treatment with AICAR and A769662, AMPK activators, decreased TLR4 and increased PPARγ protein levels in LPS-treated cells. They concluded that this anti-inflammatory effect is attributed to the inhibition of TLR4 and activation of PPARγ, which is dependent on the AMPK signaling pathway. Another study investigating diet-induced obesity on cardiac inflammatory responses showed that a high-fat diet increased the inflammatory responses and decreased glucose metabolism in the heart via suppression of AMPK activity. In this study, acute lipid infusion increased circulating fatty acids. As a result, cardiac inflammation happens by activating the TLR4 signaling pathway.^121^

 In summary, considering the studies mentioned above, it can be said that there is a relationship between APMK and TLR4. In most cases, this relationship is negative feedback.

## Expert opinion

 Inflammation is related to many diseases, together with cardiovascular complications. Innate immunity has ruled studies in CVDs. However, the significance of innate immunity results in new research regions. It might bring about novel therapeutic targets for treating inflammatory diseases. AMPK is an essential target given its co-regulatory function concerning metabolism and inflammation.

 On the other hand, broad studies have been performed on TLR modulators and their therapeutic indication in several cardiac-related disorders. Albeit, the interaction of these two critical factors in inflammatory processes needs to be clarified. The significant limitation currently facing these studies is the lack of knowledge about how effective these pathways will be. This is likely due to the complexity of cell signaling in inflammatory conditions, especially the bilateral effects of TLR’s role in either improving immune-related diseases or exacerbating them. Furthermore, depending on the context and stage of the disease, several inflammatory pathways are relevant, requiring further study in this area. Despite the various studies related to perspectives of TLR modulation,^122^ though the lack of clear understanding of the role of TLR receptors in the progression of the specific disease is one of the main hampering reasons.

 Nevertheless, a sensible therapeutic approach must be considered, which will not over-activate or -inhibit the immune defense role. The practical appearance of new immune modulator drugs by interfering AMPK pathway requires a more in-depth identification and understanding of the function and localization of different TLR receptors and various mediators in AMPK and TLR signaling pathways.

 Future studies might examine whether therapeutic interventions through AMPK related approaches can improve inflammatory disorders in immune-based disease. Furthermore, as the interference of inflammation in vast ranges of diseases spanning from organ-dependent inflammation like a CVD to systemic conditions such as autoimmune disorders and cancers; thereby any attempt to identify novel therapeutic targets within this system seems justified.

 Another use for these novel medicines will be in cardio-inflammatory diseases, which this review article, and chronic disorders with subsequent cardiovascular events such as diabetes, atherosclerosis, and hypertension. Since the footprint of inflammatory mechanisms is confirmed in the progression of all these high prevalence pathologic conditions, more profound exploration into this treatment approach can ultimately promote the development of the life sciences.

 Moreover, TLRs’ potential property in distinguishing PAMPs of diverse origin and commencing of pro-inflammatory response during various inflammatory conditions. During indication for sepsis, many cases of effectiveness in a completely different type of disease were reported, which lead the researches on their impact and indication in various conditions such as influenza-associated acute lung injury, hepatitis B, human papillomavirus, metastatic melanoma, non-small cell lung cancer and even controlling mammalian reproduction and fertilization processes.^123^

 In our opinion, novel AMPK-related TLR modulators will find application in treating different immune-mediated inflammatory and non-inflammatory disorders, especially inflammatory cardiac disease, and present an option that will be widely used in clinical practice in the future.

## Conclusion

 This review elaborates on the possible cross-talk between TLR4 and AMPK signaling in CVDs. In summary, the appropriate inflammatory response induced by TLRs is required to function both innate and adaptive immune responses to defend against damage and tissue repair. However, its inordinate signaling may be devastating and can result in cardiac disorders. Among all TLRs, TLR4 has been extensively investigated for its critical role in different myocardial inflammatory conditions. The results of several studies are explanatory of a theory that there may be a relationship between TLR4 and AMPK signaling in various inflammatory conditions. AMPK, a serine/threonine kinase, acts as a cellular energy sensor and is activated due to ATP depletion. Although AMPK signaling has a particular physiological role in the heart cells like other tissues, its importance is accentuated in stressor conditions such as myocardial ischemia. Most of the evidence from previous research highlights the anti-inflammatory benefits of the AMPK activators that point out a novel and promising therapeutic strategy in the pathophysiology of several immune-mediated inflammatory diseases. Finally, it should be noted that the correlation between AMPK and TLRs is a novel and investigational subject, with various unknown aspects, which need further research and attention.

## Acknowledgments

 The authors duly acknowledge Vice Chancellor for Research of Tabriz University of Medical Sciences, Tabriz, Iran for their support.

## Author Contributions

**Conceptualization: **Haleh Vaez.

**Data Creation: **Haleh Vaez, Hamid Soraya.

**Investigation:** Haleh Vaez, Hamid Soraya.

**Methodology:** Haleh Vaez, Alireza Garjani.

**Project administration:** Haleh Vaez.

**Supervision: **Alireza Garjani.

## Ethical Issues

 Not applicable.

## Conflict of Interest

 The authors declare that they have no competing interests.
